# Age-period-cohort analysis of pulmonary tuberculosis epidemiological trends from 2005 to 2024 and forecasts for 2035 in Sichuan Province, China

**DOI:** 10.3389/fpubh.2026.1757996

**Published:** 2026-03-24

**Authors:** Yunna Zhang, Jia Lu, Yao Ma, Ling Li, Yan Liu, Zhijun Ying, Li Zhou

**Affiliations:** Sichuan Provincial Center for Disease Control and Prevention, Chengdu, China

**Keywords:** age-period-cohort model, Bayesian age-period-cohort model, epidemiologic feature, incidence, infectious disease, pulmonary tuberculosis

## Abstract

**Background:**

Pulmonary tuberculosis (PTB) remains a significant public health challenge in Sichuan, China and globally. This study evaluated the effects of age, period, and cohort on PTB reported incidence in Sichuan and predicted the incidence from 2025 to 2035. The goal was to provide evidence to support the enhancement of PTB prevention and control strategies.

**Methods:**

PTB case and population data of Sichuan Province from 2005 to 2024 were obtained from the China Disease Prevention and Control Information System. Trends in incidence rates were analyzed using a joinpoint regression model based on natural logarithmic transformation. An age-period-cohort (APC) model was used to analyze the effects of age, period, and cohort on PTB reported incidence. A Bayesian age-period-cohort (BAPC) model was employed to forecast PTB reported incidence over the next 11 years.

**Results:**

From 2005 to 2024, a total of 1,199,575 PTB cases were reported in Sichuan Province. APC analysis revealed that the age effect peaked in the 20–24 year age group (*RR* = 285.61), while period effects showed a slow decrease. The cohort effects indicated that later cohorts had progressively lower risks. Males exhibited the highest risk at ages 20–24 years, while females peaked at ages 15–19 years. The incidence trends vary in different regions. Category 1 regions (three cities) have shown an upward trend, while Category 2 regions (remaining 18 cities) are decreasing. The BAPC model predicted that the incidence for 2035 is 55.70/100,000, exceeding the 2024 rate of 53.23/100,000.

**Conclusions:**

Our findings highlight significant gender and age disparities in PTB reported incidence, with males and individuals aged 15–24 years facing higher risks. Public health strategies for PTB eradication and prevention must be tailored to age and gender. PTB control in some regions presents substantial challenges and requires locally adapted strategies. Comprehensive enhancement of tuberculosis prevention and control efforts is essential to achieve the goal of ending epidemic.

## Background

Tuberculosis (TB) is a chronic infectious disease caused by *Mycobacterium tuberculosis*, primarily transmitted via respiratory droplets and aerosols. Primarily affecting the lungs as pulmonary tuberculosis (PTB), it is also characterized as a wasting disease. The TB epidemic represents a significant global public health and social challenge (1–3). According to the World Health Organization's Global Tuberculosis Report 2024 (4), 8.2 million people worldwide were newly diagnosed and officially notified as TB cases in 2023—the highest annual number since WHO began systematic global surveillance in the mid-1990s, indicating a potential resurgence of the global TB epidemic.

China is among the 30 high TB burden countries globally (5, 6). While significant improvements in the TB epidemic have been achieved in recent years due to sustained public health focus and increased resource allocation (7, 8), the prevalence remains concerning due to the large infected population (9, 10).

Sichuan Province, located in southwest China, exhibits a particularly high TB incidence rate (11, 12). Characterized by diverse geography, uneven regional economic development, megacities exceeding 10 million inhabitants, sparsely populated plateau regions, and disparities in public health infrastructure, Sichuan presents a complex epidemiological landscape. Notably, TB incidence in certain regions significantly exceeds the provincial average (13), creating an extremely complicated prevention and control situation. Therefore, a clear understanding of the epidemiological characteristics of PTB and the identification of high-risk factors are essential for formulating effective control strategies.

The age-period-cohort (APC) model is a statistical method based on Poisson distribution that simultaneously analyzes the influence of age, period, and cohort effects on disease incidence, overcoming the limitation of traditional modeling techniques which typically focus on only one of these factors (14–16). While widely used in cancer epidemiology, APC models have been less frequently applied to infectious diseases. Their application to chronic infectious diseases like TB holds significant potential for enhancing prevention and surveillance efforts (17–19). Previous studies predicting infectious disease incidence have primarily relied on time series models (20). By contrast, the Bayesian Age-Period-Cohort (BAPC) model incorporates the effects of age, period, and cohort simultaneously for incidence prediction, thereby revealing long-term trends in disease spread (21). Therefore, this study employed the APC model to evaluate the effects of age, period, and cohort on PTB reported incidence across different genders and regions within Sichuan Province. Furthermore, the BAPC model was applied to predict the future trend of PTB reported incidence in Sichuan Province from 2025 to 2035.

## Materials and methods

### Data source

PTB case data from 2005 to 2024 in Sichuan Province were obtained from the China Information System for Disease Control and Prevention. As PTB is a notifiable infectious disease in China, diagnosis must be confirmed based on a comprehensive assessment of clinical symptoms, signs, epidemiological history, and laboratory findings in accordance with national diagnostic criteria. All confirmed cases are required to be reported to the system by hospitals within legally mandated timeframes. To satisfy the requirements of the analytical model, which necessitated age groups with consistent 5-year intervals, the study population was restricted to individuals aged between 0 and 89 years. Participants aged 90 years and above were excluded due to the inability to form a complete 5-year interval group. Records with missing information on region or age were also excluded.

Annual population estimates by age group from 2005 to 2024 were also obtained from the same surveillance system. Age-specific predicted incidence rates were age-standardized using the WHO world standard population.

### Joinpoint regression models

Trends in incidence rates were analyzed using a joinpoint regression model based on natural logarithmic transformation to determine the presence and statistical significance of upward or downward trends. The joinpoint regression model was optimized using the Grid Search Method for model fitting, the Monte Carlo permutation test, and the Bayesian Information Criterion. The optimal model was selected based on the mean squared error (MSE), where a smaller MSE indicates better model fit. The annual percent change (APC), average annual percent change (AAPC), and their corresponding 95% confidence intervals were calculated. When the 95% CI does not include zero, the APC and AAPC are considered statistically significant, with the sign of the estimate indicating an upward or downward trend, and the magnitude reflecting the extent of the change. Estimates without statistical significance indicate a stable trend.

### The APC models

The APC models analyze epidemic characteristics using registry-based incidence data (22). The APC model is based on multiple regression and fundamentally follows a Poisson distribution. Its expression is as follows:


$$\begin{array}{l} \text{In}\left({\text{Y}}_{\text{ijk}}\right)=\mu +\alpha_{\text{i}}+\beta_{\text{j}}+\gamma_{\text{k}}+\;\varepsilon_{\text{ijk}} \end{array}$$

where Y_*ijk*_ represents the incidence rate of PTB for the *k*-th cohort in the *j*-th age group and the *j*-th period; μ is the intercept, representing the reference level of incidence under baseline age, period, and cohort; α_*i*_, β_*j*_, γ_*k*_ denote the age, period, and cohort effects; and ε_*ijk*_ represents the error or residual term. Due to the collinearity among age, period, and cohort, the intrinsic estimator (IE) method was employed to solve the APC model, thereby overcoming the issue of non-estimability of model parameters.

In the APC analysis, cases and population data were stratified into four consecutive 5-year periods (2005–2009 to 2020–2024) and 18 five-year age groups (0–89 years), generating 21 partially overlapping cohorts (1920–1924 to 2020–2024). Age-specific incidence trends were evaluated using local drift (the annual percentage change for each age group), while net drift (the overall annual percentage change) represented the composite temporal trend incorporating both period and cohort effects. Cross-sectional age effect depicts the age-specific rates observed in a given calendar period. The longitudinal age curve depicts the age effects after adjusting for period and cohort influences, which reflects differences in incidence across age groups attributable to factors such as accumulated social experience, changes in social roles, and psychological transitions. Period effects capture the impact of societal, economic, cultural, and demographic factors on incidence, whereas cohort effects represent variations in disease risk arising from differential exposure to risk factors across cohorts. The results are presented as relative risks (RRs) for period and cohort. The median period (2010–2014) and median cohort (1970–1974) were chosen as references (*RR* = 1); the choice of reference does not affect the interpretation of the estimates.

### The BAPC model

The Bayesian age-period-cohort (BAPC) model extends the classical APC framework to project future tuberculosis incidence. Full Bayesian inference was conducted using integrated nested Laplace approximations (INLA), a computationally efficient alternative to Markov chain Monte Carlo methods that provides accurate approximations of posterior marginals for latent Gaussian models without requiring convergence diagnostics. The model assumes that observed case counts follow a Poisson distribution, with the logarithm of the expected incidence rate additively decomposed into age, period, and cohort effects. Given the continuity of APC effects across adjacent time intervals, a second-order random walk (RW2) smoothing prior was employed. Assuming that the second-order smoothness structure remains stable over time, projections for the period 2025–2035 were generated by extrapolating the random walk processes for period and cohort beyond the observation window.

### Statistics

Compare the age, period, and cohort effects between different genders. The age, period and cohort effects of the regions where the incidence rate increased and decreased were analyzed respectively. PTB infection data in Sichuan China were organized using WPS software. The incidence trend was analyzed by using the Joinpoint Regression Program 5.0.0.0 Software. The APC model analysis and BAPC model analysis were performed using *R* Studio (version 4.4.1) with packages BAPC (version 0.0.36), INLA (version 24.05.011). Parameter significance was assessed by two-sided Wald χ^2^ tests with a significance level of *P* < 0.05.

## Results

### Survey of PTB reported incidence in Sichuan Province from 2005 to 2024

From 2005 to 2024, Sichuan Province reported 1,199,575 PTB cases, yielding an average annual incidence of 72.95 per 100,000 population. Incidence peaked at 112.00/100,000 in 2005 and reached its lowest point at 50.77/100,000 in 2022.

### A joinpoint regression analysis

**Figure 1:**
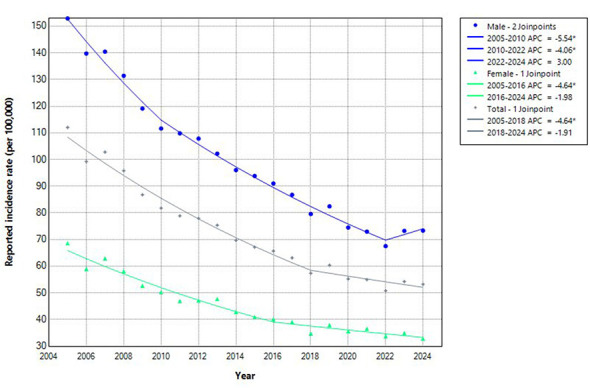
Sex-specific trends in PTB incidence in Sichuan Province, 2005−2024, estimated by joinpoint regression. Scatter points represent the observed annual incidence: stars represent the overall population, circles represent men, and triangles represent women. The solid line represents the trend fitted from the joinpoint model. Gray represents the overall; Blue indicates male; Green indicates female.

The results of the joinpoint regression analysis showed that the tuberculosis incidence rate in Sichuan Province declined by an average of 3.78% per year (95% CI: −4.29 to −3.41) from 2005 to 2024. Segmented analysis revealed an APC of −4.64% (95% CI: −6.53 to −4.17) for 2005–2018 and −1.91% (95% CI: −3.75 to −3.59) for 2018–2024 (Figure 1).

Sex-specific analysis revealed a significant overall downward trend in incidence for both males and females. Among males, the average annual percent change (AAPC) was −3.74% (95% CI: −4.25 to −3.45, *P* < 0.05). The decline was most pronounced during 2005–2010 (APC = −5.54%, 95% CI: −8.14 to −4.23, *P* < 0.05), slowed to −4.06% (95% CI: −6.39 to −2.82, *P* < 0.05) during 2010–2022, and was followed by a non-significant increase of 3.00% per year (95% CI: −3.06 to 6.08, *P* > 0.05) during 2022–2024. In females, the AAPC was −3.53% (95% CI: −4.11 to −3.03, *P* < 0.05). The decrease was significant during 2005–2016 (APC = −4.64%, 95% CI: −8.07 to −3.85, *P* < 0.05), while the estimated change during 2016–2024 (APC = −1.98%, 95% CI: −3.53 to 3.96, *P* > 0.05) did not reach statistical significance (Figure 1).

**Figure 2:**
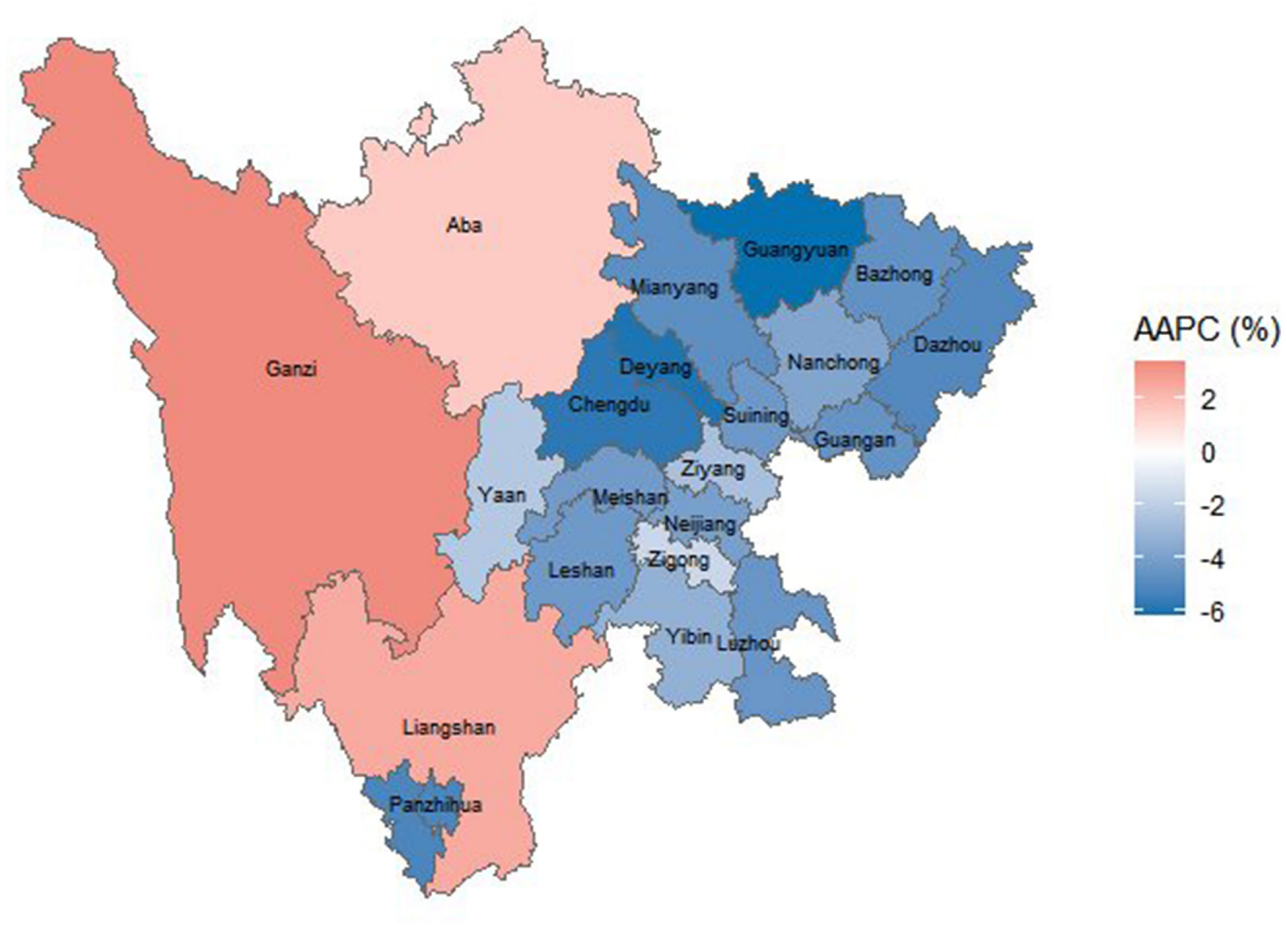
Spatial distribution of PTB incidence trends across 21 cities in Sichuan Province, 2005–2024. Colors represent the average annual percent change (AAPC): orange indicates increasing trends (AAPC > 0); blue indicates decreasing trends (AAPC < 0). Darker shades correspond to larger absolute AAPC values.

Stratified analysis across the 21 cities in Sichuan Province revealed that three cities—Ganzi, Liangshan, and Aba—exhibited a significant increasing trend (AAPC > 0, *P* < 0.05), in contrast to the overall provincial pattern; these were classified as Category 1 regions. The remaining 18 cities showed a significant decreasing trend (AAPC < 0, *P* < 0.05) and were designated as Category 2 regions (Figure 2).

### An age-period-cohort model analysis of PTB reported incidence

From 2005 to 2024, the net drift in the incidence of PTB in the whole population, all period *RR*, all cohort *RR* and all local drifts were statistically significant (*P* < 0.05), and also statistically significant in terms of gender and region (*P* < 0.05; Table 1), suggesting that the incidence of PTB was influenced by age, period and cohort effects.

**Table 1:** Wald tests of APC model parameters of pulmonary PTB in Sichuan Province, 2005–2024.

**Group**	**Model parameter**	**χ^2^**	**df**	***P*-value**
Overall	Net drift = 0	226.44	1	< 0.001
	All age deviations = 0	319.10	16	< 0.001
	All period deviations = 0	1.18	2	0.55
	All cohort deviations = 0	43.80	19	< 0.001
	All period *RR* = 1	238.31	3	< 0.001
	All cohort *RR* = 1	313.80	20	< 0.001
	All local drifts = net drift	43.71	18	< 0.001
Male	Net drift = 0	242.48	1	< 0.001
	All age deviations = 0	269.07	16	< 0.001
	All period deviations = 0	1.09	2	0.58
	All cohort deviations = 0	30.85	19	0.04
	All period *RR* = 1	254.19	3	< 0.001
	All cohort *RR* = 1	332.21	20	< 0.001
	All local drifts = net drift	30.79	18	0.03
Female	Net drift = 0	172.73	1	< 0.001
	All age deviations = 0	445.64	16	< 0.001
	All period deviations = 0	1.93	2	0.38
	All cohort deviations = 0	78.53	19	< 0.001
	All period *RR* = 1	183.66	3	< 0.001
	All cohort *RR* = 1	266.11	20	< 0.001
	All local drifts = net drift	78.50	18	< 0.001
Category 1 regions	Net drift = 0	12.67	1	< 0.001
	All age deviations = 0	838.19	16	< 0.001
	All period deviations = 0	7.62	2	0.02
	All cohort deviations = 0	182.94	19	< 0.001
	All period *RR* = 1	21.21	3	< 0.001
	All cohort *RR* = 1	237.42	20	< 0.001
	All local drifts = net drift	181.93	18	< 0.001
Category 2 regions	Net drift = 0	283.21	1	< 0.001
	All age deviations = 0	233.14	16	< 0.001
	All period deviations = 0	0.38	2	0.83
	All cohort deviations = 0	27.66	19	0.09
	All period *RR* = 1	295.24	3	< 0.001
	All cohort *RR* = 1	418.22	20	< 0.001
	All local drifts = net drift	27.36	18	0.07

### Time trends in PTB reported incidence

In Sichuan Province, the Net Drift of PTB reported incidence was −4.40%. Local Drifts were negative across all age groups except for the 10–14 year age group. Among males, the Net Drift was −4.76%, with negative Local Drifts observed in all age groups. Among females, the Net Drift was −3.70%, with negative Local Drifts in all age groups except the 5–9 and 10–14 year groups.

Category 1 regions demonstrated a positive Net Drift (0.88%). Positive Local Drifts were observed in the 0–29 years, 35–39 years, and ≥70 years age groups. Notably, the 10–14 year age group showed the highest increase (Local Drift = 8.07%). Conversely, Category 2 regions had a negative Net Drift (-6.19%), with negative Local Drifts observed in all age groups (Table 2).

**Table 2:** Local drifts and net drifts from age-period-cohort analyses of PTB reported incidence in Sichuan province, 2005–2024.

**Age**	**Overall**	**Gender**		**Region**	
		**Male**	**Female**	**Category 1**	**Category 2**
Net drifts	−4.404	−4.7601	−3.6995	0.879	−6.1855
**Local drifts**					
0–4	−3.43	−4.19	−2.32	2.31	−12.36
5–9	−0.49	−1.98	1.4	6.96	−11.79
10–14	1.08	−0.13	2.45	8.07	−5.77
15–19	−1.41	−2.24	−0.32	6.07	−5.30
20–24	−3.6	−3.92	−3.03	3.72	−5.96
25–29	−4.65	−5.04	−3.97	0.57	−6.02
30–34	−4.78	−5.3	−3.88	−0.07	−6.26
35–39	−5.09	−5.58	−4.16	0.71	−6.91
40–44	−6.72	−6.96	−6.02	−0.28	−8.39
45–49	−5.19	−5.29	−4.67	−1.13	−6.03
50–54	−4.43	−4.4	−4.28	−0.99	−4.97
55–59	−4.58	−4.36	−4.94	−0.17	−5.09
60–64	−5.72	−5.43	−6.25	−0.31	−6.23
65–69	−5.51	−5.36	−5.54	−0.03	−5.97
70–74	−4.69	−5.05	−3.6	0.28	−5.14
75–79	−3.99	−4.87	−1.74	0.73	−4.41
80–84	−3.23	−4.7	−0.14	0.42	−3.53
85–89	−5.27	−7.02	−2.5	−4.35	−5.39

### Age effect of PTB reported incidence

**Figure 3:**
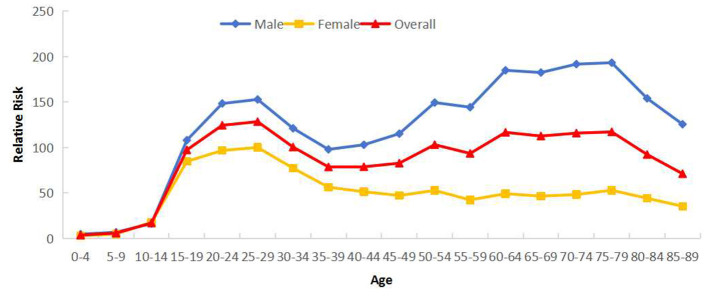
Cross age effects of reported incidence of PTB in Sichuan Province stratified by gender (male and female).

Cross-sectional age effects reveal that PTB reported incidence in Sichuan Province (2005–2024) exhibited a bimodal distribution across age groups. The relative risk (*RR*) gradually increased with age, peaking first in the 15–29 year group, declining through ages 35–39, then rising again to form a second peak in the 65–79 year group. All genders showed this dual-peak pattern, though males demonstrated higher RRs than females across all ages. While males exhibited a higher second peak than their first peak, females showed a lower second peak relative to their initial peak (Figure 3).

**Figure 4:**
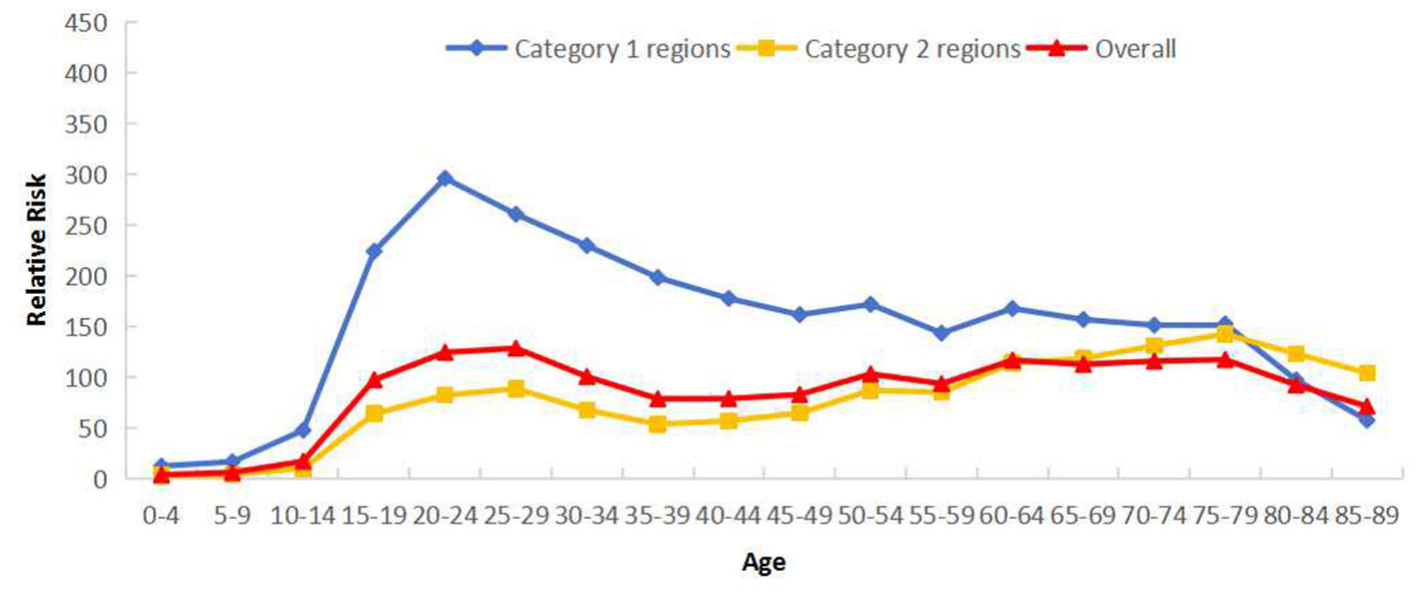
Cross age effects of reported incidence of PTB in Sichuan Province stratified by regions: Category 1 (areas with rising incidence) and Category 2 (areas with falling incidence).

Category 2 regions mirrored Sichuan's overall bimodal age trend. Conversely, Category 1 regions displayed only one significant incidence peak in the 15–34 year group, with substantially higher RRs than observed in Category 2 regions (Figure 4).

**Figure 5:**
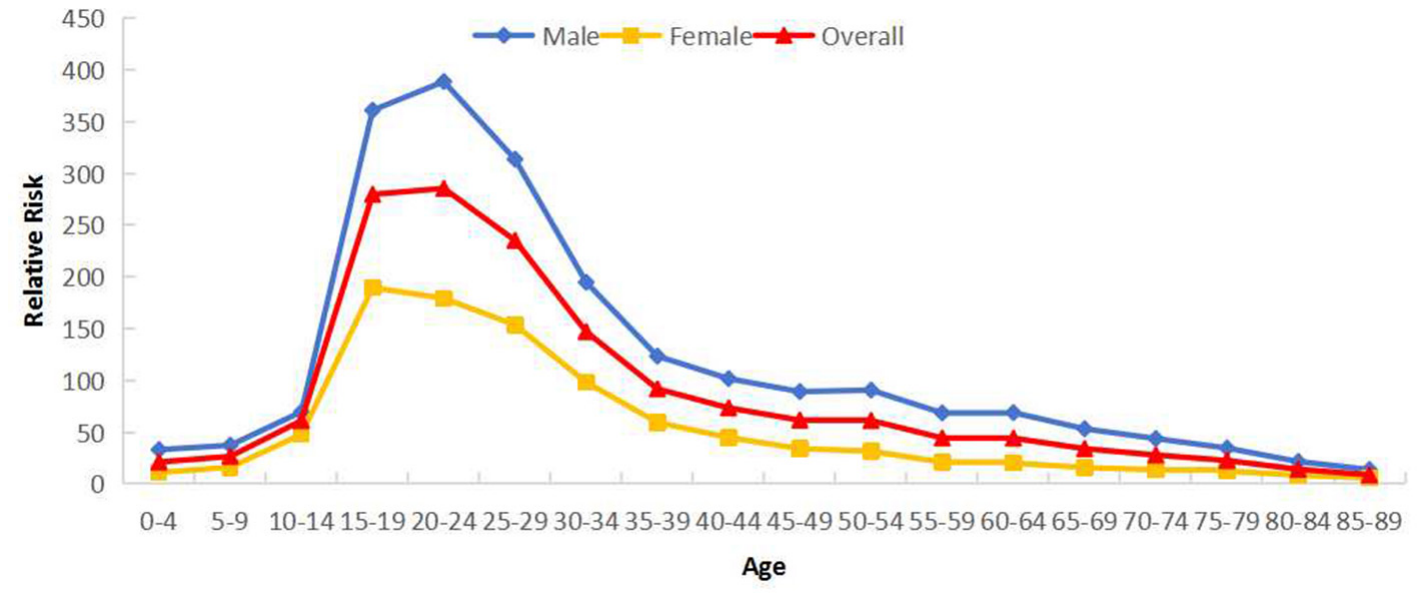
Long age effects of reported incidence of PTB in Sichuan Province stratified by gender (male and female).

**Figure 6:**
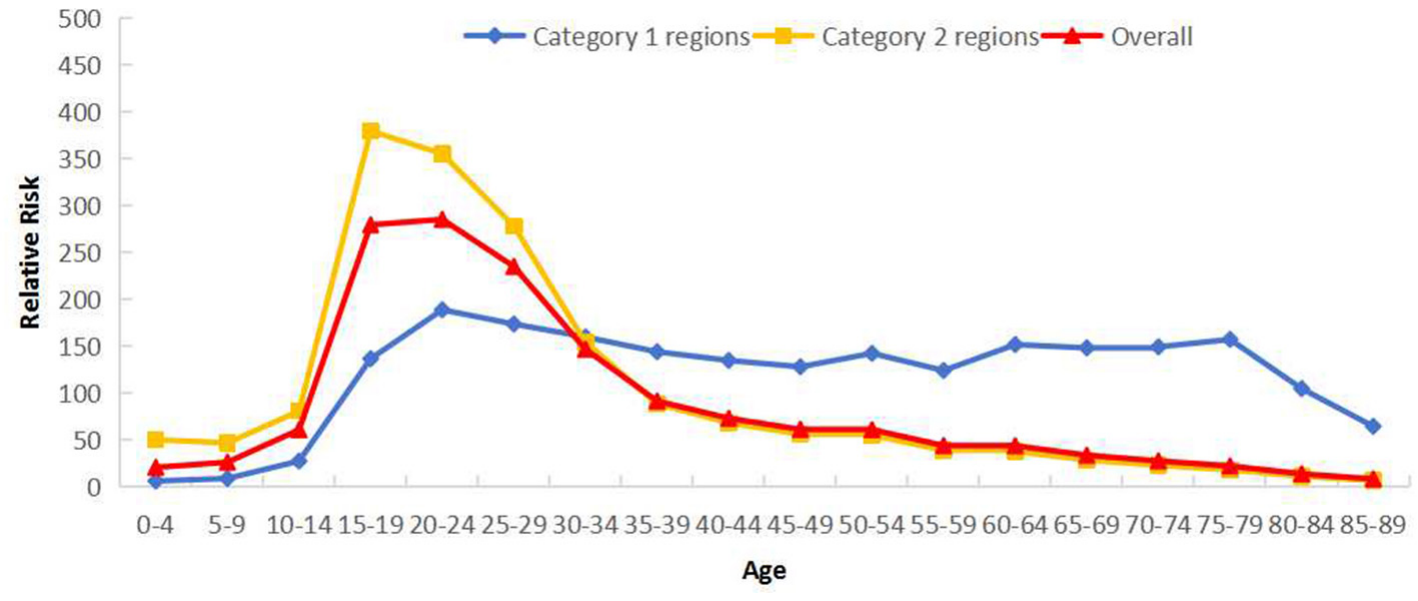
Long age effects of reported incidence of PTB in Sichuan Province stratified by regions: Category 1 (areas with rising incidence) and Category 2 (areas with falling incidence).

As shown in long age effects, PTB reported incidence in Sichuan showed a unimodal distribution that increased with age. *RR* was low at 0–9 years, increased significantly thereafter, peaked at 20–24 years (*RR* = 285.61), then declined rapidly. Males exhibited higher RRs than females across all ages. The highest *RR* for males occurred at 20–24 years, while females peaked at 15–19 years (Figure 5). Regionally, Category 2 regions showed peak *RR* at 15–29 years, exceeding Category 1 regions' peak. Comparably high RRs were observed across the 15–79 age range in Category 1 regions (Figure 6).

### Period effect of PTB reported incidence

**Figure 7:**
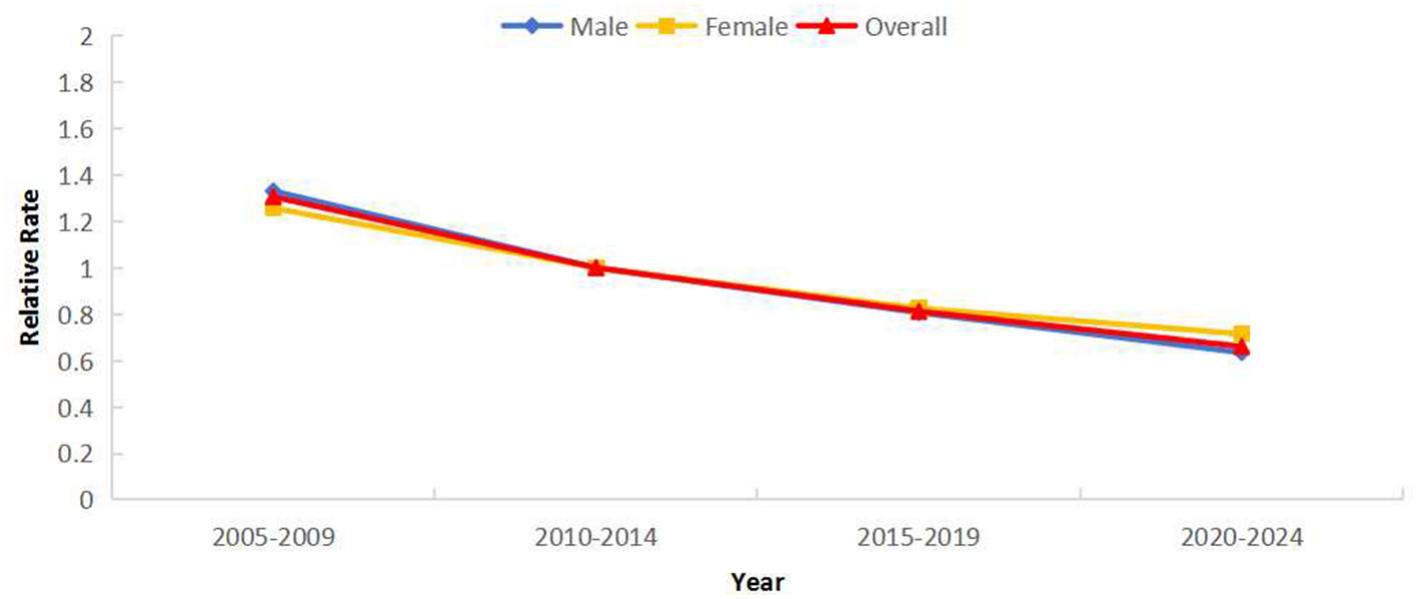
Period effects of reported incidence of PTB in Sichuan Province stratified by gender (male and female).

**Figure 8:**
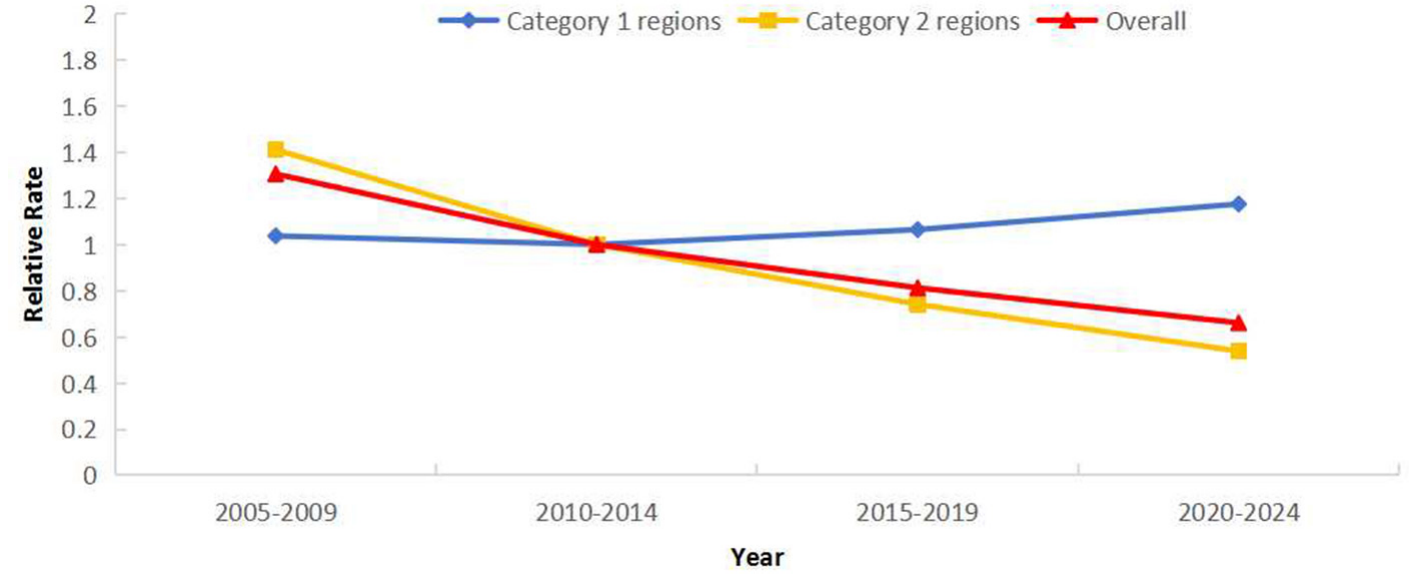
Period effects of reported incidence of PTB in Sichuan Province stratified by regions: Category 1 (areas with rising incidence) and Category 2 (areas with falling incidence).

*RR* demonstrated a gradual temporal decline. Using 2010–2014 as reference, risk was highest in 2005–2009 (*RR* = 1.31) and lowest in 2020–2024 (*RR* = 0.66). All genders showed declining trends, with males exhibiting a greater decline than females (Figure 7). Notably, *RR* increased in Category 1 regions, reaching 1.17 times the reference period during 2020–2024 (Figure 8).

### Cohort effect of PTB reported incidence

**Figure 9:**
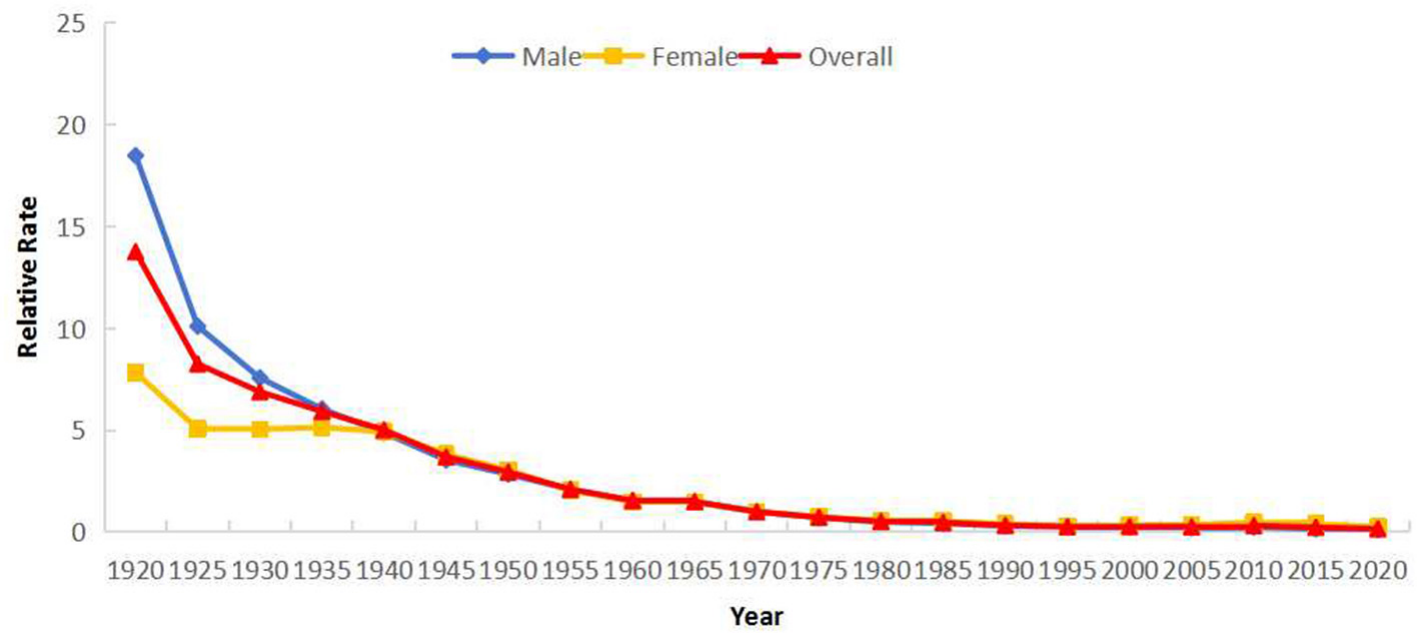
Cohort effects of reported incidence of PTB in Sichuan Province stratified by gender (male and female).

**Figure 10:**
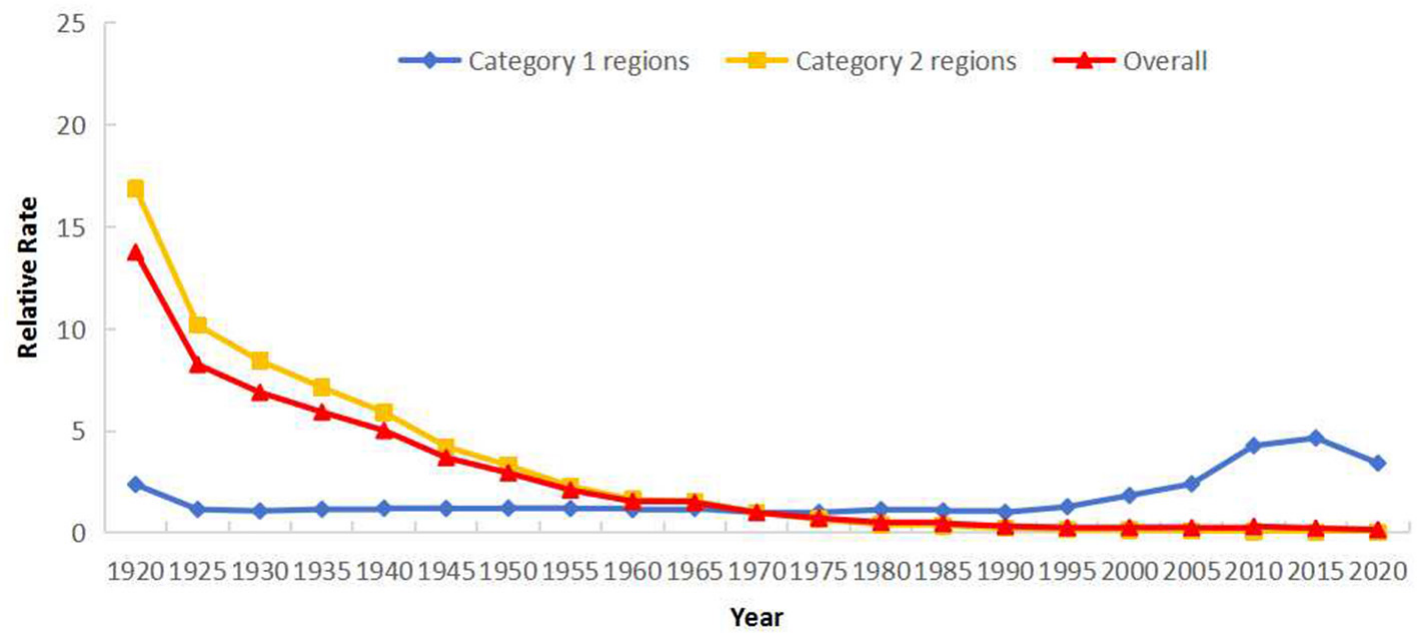
Cohort effects of reported incidence of PTB in Sichuan Province stratified by regions: Category 1 (areas with rising incidence) and Category 2 (areas with falling incidence).

Cohort effects indicated decreasing risk with later birth years. Using the 1970–1974 cohort as reference (*RR* = 1), *RR* declined from 13.75 (1920–1924 cohort) to 0.16 (2020–2024 cohort). Post-1985 cohorts had *RR*s below 0.50. Gender-specific patterns mirrored overall trends (Figure 9). Category 2 regions showed higher risk before 1970, while Category 1 regions exhibited higher risk thereafter. Cohorts born after 1995 in Category 1 regions demonstrated significantly elevated risk (Figure 10).

### BAPC model prediction of disease trends and incidence

**Figure 11:**
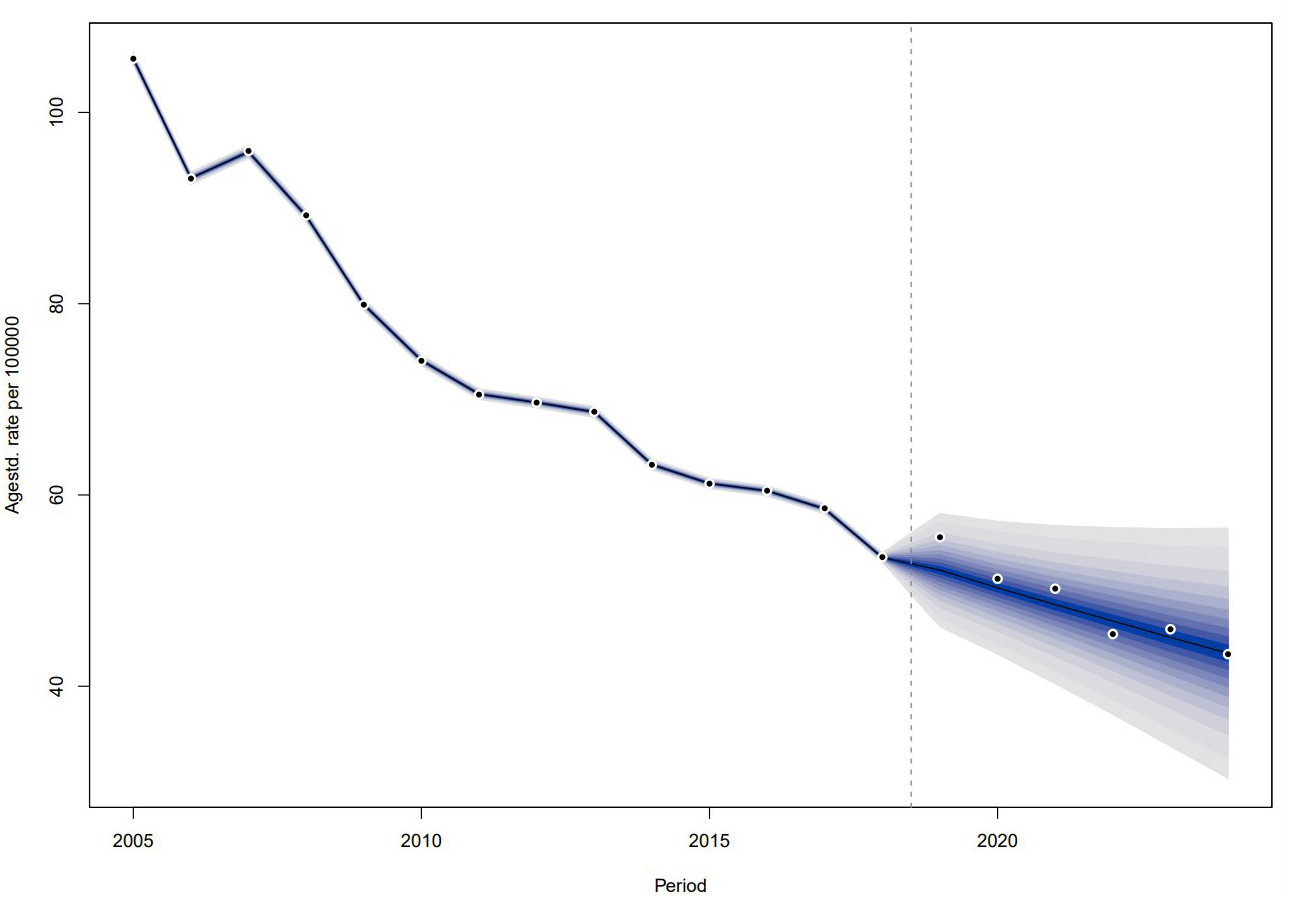
Internal validation of the BAPC model for PTB incidence in Sichuan Province (2019–2024). The model was trained on data from 2005 to 2018 to predict incidence for 2019–2024. The solid line represents the predicted incidence and the dots represents the actual reported incidence.

To evaluate the predictive performance of the BAPC model, we used PTB incidence data from 2005 to 2019 to forecast incidence for the period 2020–2025, and compared the predicted values with the actual incidence rates. The results showed that the mean absolute percentage error (MAPE) was 3.51%, and all actual values fell within the 95% confidence intervals (Figure 11).

**Figure 12:**
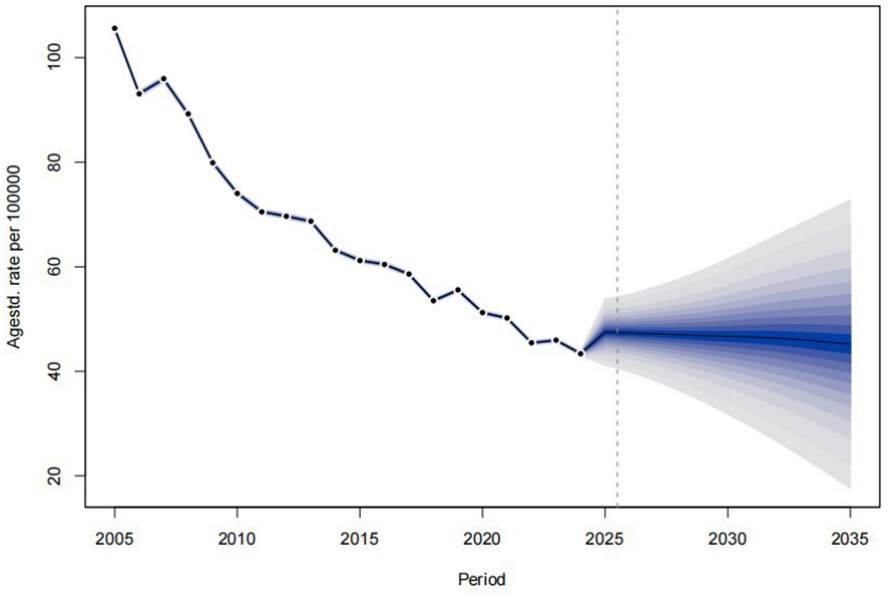
BAPC model predicts the trend of incidence of PTB in Sichuan Province from 2025 to 2035(color intensity corresponds to probability density; darker shades indicate a higher probability of incidence values occurring).

The BAPC model predicted PTB reported incidence in Sichuan (2025–2035), indicating an initial increase in 2025 followed by gradual decline (Table 3, Figure 12). Projected incidence for 2035 is 55.70/100,000, exceeding the 2024 rate (53.23/100,000).

**Table 3:** Predicted incidence of PTB in Sichuan Province from 2025 to 2035.

**Year**	**Predicted incidence (per 100,000)**	**95% Cis (per 100,000)**
2025	55.33	44.62~66.04
2026	55.21	44.22~66.20
2027	55.15	43.75~66.55
2028	55.14	43.16~67.12
2029	55.22	42.46~67.98
2030	55.38	41.62~69.14
2031	55.57	40.61~70.53
2032	55.73	39.39~72.07
2033	55.75	37.87~73.63
2034	55.71	36.09~75.33
2035	55.70	34.11~77.29

## Discussion

Since 2005, the reported incidence of PTB in Sichuan Province has shown a declining trend. This decline coincides with sustained governmental interventions in tuberculosis control (23, 24). The decline rate slowed after 2018, with male incidence increasing post-2022. This partial reversal may be related to China's 2017 diagnostic criteria revision, which broadened the case definition to include tracheal, bronchial, and pleural tuberculosis and incorporated newer diagnostic technologies (25). Such changes in diagnostic criteria can artificially increase reported incidence, making it difficult to distinguish between true epidemiological changes and surveillance-related artifacts. Additionally, intensified screening of high-risk groups in Sichuan has enhanced case detection, which may have contributed to higher reported rates in recent years. Nevertheless, potential incidence rebound remains a concern.

Discrepancies between cross-sectional and longitudinal age-specific rates indicate potential confounding in descriptive analyses (26). The Age-Period-Cohort (APC) modeling approach helps mitigate such bias and provides a more accurate representation of epidemiological patterns. Cross-sectional analysis indicated dual incidence peaks (15–29 and 60–79 years), whereas APC modeling identified only one age-driven peak (15–29 years). This finding suggests a strong cohort effect, with elevated risk in 60–79-year-olds likely attributable to earlier birth cohorts rather than aging *per se*. One plausible explanation is that early-life exposure to adverse environmental factors, including childhood malnutrition, may compromise immune development, increasing susceptibility to respiratory infections and latent pathogen re-emergence in later life (27).

Sichuan demonstrates low PTB reported incidence among children aged 0–10 years, contrasting with US data showing peak lifetime incidence in this age group (28). This discrepancy may reflect China's inclusion of BCG vaccination in the national immunization program since 1978, which has been associated with substantially reduced pediatric infection risk (29). However, BCG offers limited adult protection, which may partially explain the significant incidence increase at 15–29 years. Notably, different from other age groups, the 10–14-year age group shows a positive annual change percentage, indicating a rising trend. Enhanced school-based screening programs in recent years may have increased case detection in this age group, contributing to the observed trend. Therefore, prioritizing school tuberculosis control through comprehensive prevention strategies—including new student physical examinations, close contact screening, and source identification—remains a public health priority.

Period effects reflect the impact of temporal factors on disease incidence and mortality across all age groups or cohorts. Notably, changes in diagnostic criteria (e.g., the 2017 revision that expanded case definitions and incorporated new tests) can create artificial shifts in reported incidence, which are captured as part of the period effect. In our analysis, despite a potential upward pressure on reported cases from such revisions post-2017, the estimated period effect relative risks (RRs) showed a significant overall declining trend. This observation suggests that the underlying impact of continuous public health system improvements and advancing medical standards may have been sufficient to outweigh any artificial inflation due to enhanced detection, potentially indicating genuine strengthening of tuberculosis control over time (30).

The cohort effects demonstrated decreasing TB incidence RR with later cohorts, indicating reduced risk for more recently born individuals—a pattern consistent with existing literature. This trend may be associated with China's evolving tuberculosis control strategies across different eras: initial measures included nationwide BCG vaccination, large-scale active case-finding, and comprehensive chemotherapy to rapidly contain TB transmission. The full implementation of the DOTS strategy in the 1990s further consolidated these gains. In recent years, China has focused on establishing a new “trinity” tuberculosis prevention and control service system composed of disease prevention and control institutions, designated tuberculosis medical institutions, and primary-level medical and health institutions. However, it is important to recognize that this cohort effect likely reflects not only targeted public health interventions but also broader socioeconomic developments, including improved nutrition, better living conditions, and higher educational attainment, all of which are known to influence tuberculosis epidemiology (31).

Given established gender differences in TB epidemiology, we conducted stratified analyses by sex (32). The APC model demonstrated consistently higher male risk across all ages—a pattern observed in Japan, Canada, and Australia where male TB rates exceed female rates in most age groups (33, 34). This disparity is likely multifactorial. In the studied settings, where significant differences in social engagement or healthcare access between men and women are unlikely, biological mechanisms and health-related behaviors may play more prominent roles (35). Evidence suggests that female hormones may have a protective effect against TB infection and development. In addition, detrimental behaviors more prevalent among males, particularly smoking and alcohol abuse, represent established TB risk factors that may increase susceptibility. Consequently, males constitute a crucial target demographic for TB control strategies, with smoking and alcohol reduction interventions potentially contributing to transmission reduction (36, 37), though the effectiveness of such interventions would need to be evaluated through rigorous study designs.

Given Sichuan's distinct regional characteristics, we analyzed Category 1 and Category 2 regions separately. Category 1 regions, populated primarily by Tibetan and Yi ethnic groups, demonstrate significantly higher TB incidence than Category 2 regions, with rates continuing to rise. These regions face substantial challenges including inadequate health resource allocation, underdeveloped service technologies, and limited public awareness, creating a severe epidemic control situation. Age-effect analysis revealed lower incidence risk among 15–19-year-olds but consistently higher risk among those over 20 in Category 1 vs. Category 2 regions. This differential pattern may stem from educational limitations, delayed healthcare-seeking behaviors, and insufficient medical resources in Category 1 regions, potentially contributing to postponed case identification (38). Period effects indicated increasing disease risk in Category 1 regions, while cohort effects showed significantly elevated risk among post-1995 cohorts. These trends may reflect later TB control program initiation in these regions, as well as intensified recent screening efforts, which could have increased case detection rates.

We employed the BAPC model to project the future incidence of pulmonary tuberculosis in Sichuan Province. First, the predictive performance of the model was evaluated through internal validation, which demonstrated good agreement between predicted and observed values (MAPE = 3.51%, with all actual values falling within the 95% confidence intervals). Subsequently, using incidence data from 2005 to 2024, we projected tuberculosis incidence for the period 2025–2035. The results indicate that the projected incidence rate in Sichuan Province in 2035 remains higher than that in 2024, suggesting that under current trends, the trajectory may fall short of the ambitious targets set by the World Health Organization's “End TB Strategy,” which aims for tuberculosis elimination by 2035. Achieving a long-term, rapid reduction in the TB burden will likely require enhanced efforts, including early detection and treatment of active TB, as well as targeted interventions for high-risk populations (39, 40). It should be emphasized that the prediction results primarily rely on the extrapolation of trends in age, period, and cohort effects from historical data, assuming that these patterns will continue in the future. However, the BAPC model cannot incorporate potential major breakthroughs in prevention and control, policy shifts, or social changes that may occur in the future. The model's core value lies in highlighting that if the current level of prevention and control efforts is maintained, achieving the goal of ending tuberculosis by 2035 may be challenging. The actual future trajectory of the epidemic will ultimately depend on the intensity and effectiveness of future intervention measures, as well as on broader societal changes that cannot be predicted from historical data alone (40–42).

This study has certain limitations. First, since the data are derived from reported PTB cases, the incidence rate may be underestimated due to variations in the quality of tuberculosis diagnosis and reporting. Second, given that data are available only for the past 20 years, it is not feasible to estimate trends or cohort effects for earlier periods. Third, since this is a population-based study, ecological fallacies may potentially arise, requiring cautious interpretation of the findings. Fourth, while the APC model effectively identifies associations, it cannot establish causality, and the interpretations presented in this discussion should be viewed as hypotheses requiring confirmation through other study designs.

## Conclusion

Our findings reveal significant gender and age disparities in PTB reported incidence, with males and individuals aged 20–24 years facing elevated risks. Consequently, public health strategies for TB elimination must be tailored to age and gender profiles. Notably, different types of regions exhibit varying age, period, and cohort effects. In minority areas, period effects increased, while post-1995 cohorts demonstrated substantially higher risk, particularly among 15–79-year-olds. TB control in minority areas presents formidable challenges requiring context-specific approaches.

Furthermore, projections suggest that the TB burden in Sichuan will increase in the coming years. Comprehensive enhancement of TB control quality is essential for achieving the UN Sustainable Development Goals and ending the TB epidemic. We anticipate these findings will inform TB elimination efforts in China and globally, enabling development of targeted public health strategies to accelerate progress toward epidemic containment.

## Data Availability

The original contributions presented in the study are included in the article/supplementary material, further inquiries can be directed to the corresponding author.
